# Supplementary material for: Multilevel determinants of illness since birth among infants aged 5–9 weeks in Ethiopia: Evidence from the 6-week follow-up of the PMA-ethiopia longitudinal survey (2021–2023)
Source: PLoS One. 2026 Feb 26;21(2):e0342776. doi: 10.1371/journal.pone.0342776 (PMC12944765; doi:10.1371/journal.pone.0342776)
Supplement: Attachment — Submitted filename: Response to Reviewers.docx [file pone.0342776.s001.docx]

**Dear Editors and Reviewers,**

We sincerely thank you for your thorough review of our manuscript and for your constructive and insightful comments. We greatly appreciate the time and effort you devoted to evaluating our work. Your comments and suggestions have significantly improved the clarity, methodological rigor, and overall quality of the manuscript. We have carefully addressed each comment and revised the manuscript accordingly. Below, we provide a detailed, point-by-point response to all comments, with specific page and line numbers indicating where changes have been made.

**Editors’ Comment**

1. Please upload a Response to Reviewers letter which should include a point-by-point response to each of the points made by the Editor and/or Reviewers.

**Authors’ Response:**
We have prepared and uploaded this **Response to Reviewers** document, providing a clear point-by-point response to all editorial requests. This document has been uploaded using the file type **“Response to Reviewers”**, as instructed.

2. Your ethics statement should only appear in the Methods section of your manuscript.

**Authors’ Response:**
We have revised the manuscript so that the **ethics statement appears only in the Methods section**. Any duplicate or misplaced ethics statements previously appearing in other sections have been removed. The ethics statement is now clearly presented under the Methods section in accordance with PLOS ONE guidelines.

Please kindly review the revisions made in the updated manuscript on pages 10–11, specifically lines 198–203.

3. Additional clarification is required to ensure adherence to the PLOS ONE Data Availability policy, particularly regarding third-party data access, replicability, and access privileges.

**Authors’ Response:**

Thank you for this comment. We have revised the Data Availability Statement to provide explicit clarification regarding third-party data access, replicability, and access privileges, in full accordance with the PLOS ONE Data Availability policy.

The data used in this study are third-party data obtained from the Performance Monitoring for Action (PMA)-Ethiopia Longitudinal Survey (2021–2023). At the time the authors accessed the data, PMA had completed its transition to an open-access data-sharing model. The datasets were freely downloaded from the PMA website, which provides direct public access to datasets hosted in the Johns Hopkins University (JHU) Data Repository, without any requirement for registration, institutional affiliation, or data access requests.

To address the specific points raised:

a) **How others can access the minimal datasets:**

**Authors’ Response:** All interested researchers can access the exact datasets used in this study by visiting the PMA data portal at: <https://www.pmadata.org/data/available-datasets>. From this page, users can directly download the PMA-Ethiopia Longitudinal Survey datasets (2021–2023), which are publicly available through the JHU Data Repository.

b) **Replicability of the study findings:**

**Authors’ Response:** Interested researchers can fully replicate the findings of this study by directly obtaining the same PMA-Ethiopia Longitudinal Survey datasets from the PMA website and following the methods described in the Methods section of the manuscript. The datasets provided by PMA constitute the minimal dataset necessary to reproduce all analyses reported in the study.

c) **Special access privileges:**

**Authors’ Response:** The authors confirm that they did not receive any special access privileges. The data were accessed under the same conditions available to all users, and no additional permissions, approvals, or restricted access were required.

The manuscript has been updated accordingly, and the revised Data Availability Statement now clearly reflects the open-access nature of the data, the absence of access restrictions, and the replicability of the study.

Please refer to the revised text in the updated manuscript on page 30, lines 576–580.

**Reviewer’s Comments**

1. *In the Abstract, the results section mentions uniformity, but some sentences use percentages while others use time; it is better to maintain uniformity.*

**Authors’ Response:**
Thank you for this observation. We have revised the Abstract to maintain consistency in reporting results by using percentages throughout. Kindly review the revised text on page 2, lines 27–31.

2. *Line 54–56 should include citations.*

**Authors’ Response:**

Thank you for your insightful comment. We have added the appropriate citations to support these statements. Please review on page number 4, lines 54 and 56.

3. *The research gap or statement of the problem was not identified properly.*

**Authors’ Response:**
We appreciate this comment and agree that the initial version did not sufficiently articulate the research gap. The Introduction has been revised to clearly define the gap and justify the study’s focus. Please review the revised text on page 4, lines 60–66.

4. *The dependent variable section (line 122) needs paraphrasing and clarification. If an infant suffered from multiple diseases, how was it categorized? Suggest making the dependent variable categorical (single, double, multi-disease).*

**Authors’ Response:** Thank you for this valuable suggestion. Thank you for this important comment. We have revised the dependent variable section to improve clarity and explicitly explain how infants with multiple symptoms were classified. Our outcome variable was defined as a binary measure (“the presence of any illness since birth: yes/no”) because the data capture maternal reports of symptoms rather than clinically diagnosed diseases. As such, it is not possible to reliably distinguish or count discrete disease entities (e.g., single, double, multiple diseases etc...), since multiple symptoms may reflect a single underlying condition or overlapping clinical presentations. Categorizing infants into single, double, or multi-disease groups would therefore risk misclassification and introduce measurement error. Consistent with prior population-based neonatal studies using symptom-based surveillance, we classified an infant as “ill” if the mother reported any one or more symptoms since birth, regardless of the number of symptoms reported (Delele, Biks et al. 2021, Chilot, Belay et al. 2022). This approach aligns with our study objective of estimating the overall burden of early infant morbidity and identifying individual and community level determinants of illness, rather than assessing disease-specific or symptom counts. We have clarified this rationale in the Methods section and revised the wording of the dependent variable definition to explicitly state that infants with multiple reported symptoms were categorized as “ill” under the binary outcome definition.

5. *There is repetition between the dependent variable section and the operational definition; these should be merged.*

**Authors’ Response:** We agree with this comment. The repetitive content has been removed, and the dependent variable description has been merged with the operational definition to improve clarity and conciseness.

6. *Readers want to know how the wealth quintile (poor to richest) was constructed.*

**Authors’ Response:** Thank you for highlighting this point. We have expanded the Methods section to describe the construction of the wealth quintile variable. The variable was obtained directly from the PMA-Ethiopia dataset and constructed using an asset-based wealth index generated through principal component analysis, following standard DHS methodology. Kindly review the revised text on page 8, lines 152–156.

7. *Which variables were included in the analysis?*

**Authors’ Response:** We appreciate this comment. A clear list of all independent variables (individual and community-level variables) has been explicitly stated in the Methods section (Please refer to page 7-8, lines 131–144). In the final analysis, the following variables were included, as described in the manuscript on page 21, lines 409–415. Based on the bivariable analysis, variables with a p-value ≤ 0.25 that were included in the final multivariable multilevel binary logistic regression model comprised maternal age, gender of the newborn, religion, and region; wealth quintile; number of antenatal care visits; receiving tetanus injection during pregnancy; consuming medication for intestinal worms during ANC; and having weight measured as part of ANC. Additionally, factors such as the number of fetuses in the current pregnancy, the mother's emotional response to the pregnancy, the partner’s reaction, birth preparedness and complication readiness, cesarean delivery, and the presence of complications during pregnancy, childbirth, and the postpartum period were all taken into account. Furthermore, initiation of breastfeeding within one hour, household use of insecticide-treated bed nets, cooking fuel type, and sources of drinking water were also included in the final model.

8. *Why was the same variable used in community poverty status?*

**Authors’ Response:** Thank you for this question. We have clarified that community poverty status was derived by aggregating household wealth indices at the cluster level, which differs conceptually from individual household wealth. This explanation has been added to the Methods section. Please refer to the revised text on page 9, lines 168–176.

9. *In Table 9, some variables are significant at 0.25 instead of 0.05.*

**Authors’ Response:** We appreciate this observation. We clarified in the Methods section that a p-value of 0.25 was used as a screening criterion for multivariable analysis, while statistical significance in the final model was evaluated at p < 0.05. This clarification has also been added to the table notes. This information is provided in the Data Analysis subsection of the manuscript (kindly review page 10, lines 186–187 and line 195).

10. *The table sentence structures are not uniform.*

**Authors’ Response:** Thank you for pointing this out. All table titles, captions, and notes have been revised to ensure consistent sentence structure and formatting throughout the manuscript. The revision has been updated and incorporated at the end of the manuscript, following the references.

11. *Sampling design and sample collection across different regions need to be clearly presented.*

**Authors’ Response:** We appreciate this important comment. The sampling design and data collection procedures, including regional representation, have now been clearly described in the Methods section (kindly review the revision on Page number 06; line number 100-109).

12. *Also, intimate partner violence was mentioned in the results but not discussed.*

**Authors’ Response:**

Thank you for this question. Intimate partner violence was included in the bivariable analysis but was not significantly associated with the outcome (COR = 1.23; 95% CI: 0.50–3.04; p = 0.65). As it did not meet the predefined inclusion criterion (p ≤ 0.25), it was excluded from the final multivariable model and therefore not discussed further.

13. *Too many tables are used; only important tables should remain in the manuscript body.*

**Authors’ Response:** Thank you for this comment. In response, we have relocated the tables to the end of the manuscript, following the references.

Once again, we sincerely thank you for your valuable feedback, which has greatly strengthened the manuscript.

**Sincerely,**
*The Authors*
